# Supplementary figures and images for: Amniotic fluid reduces liver fibrosis by attenuating hepatic stellate cell activation
Source: Stem Cells Transl Med. 2025 Jul 7;14(7):szaf026. doi: 10.1093/stcltm/szaf026 (PMC12232411; doi:10.1093/stcltm/szaf026)

Supplemental Figure 2

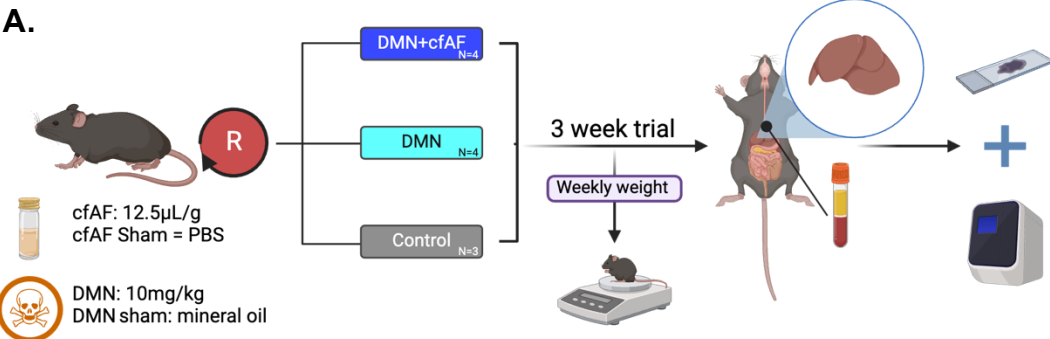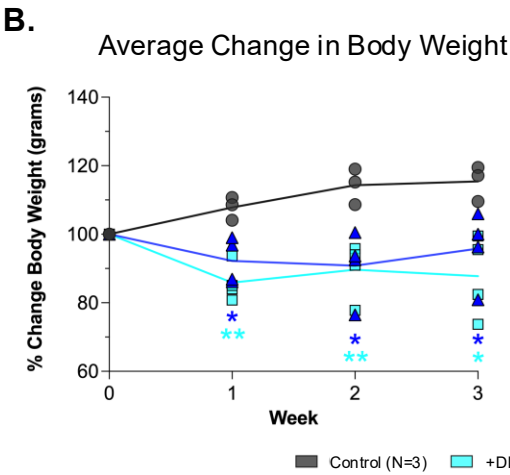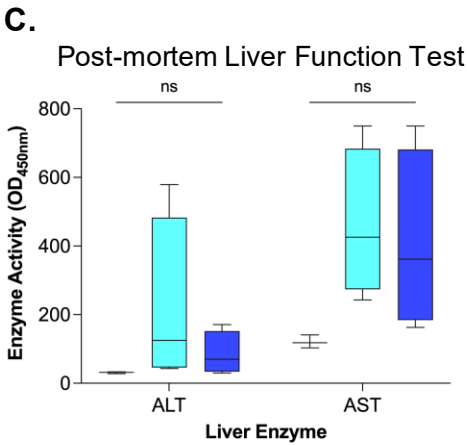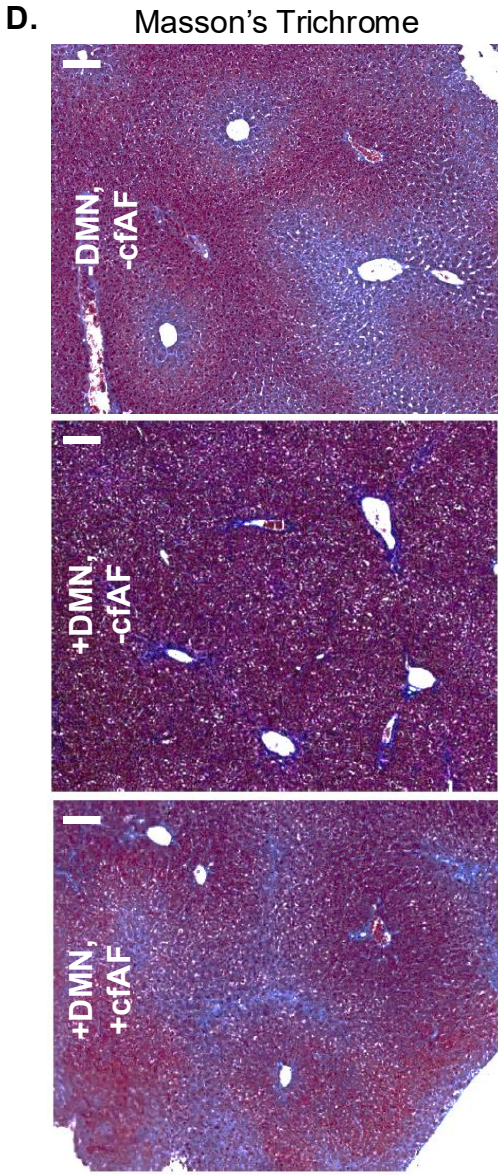

A.

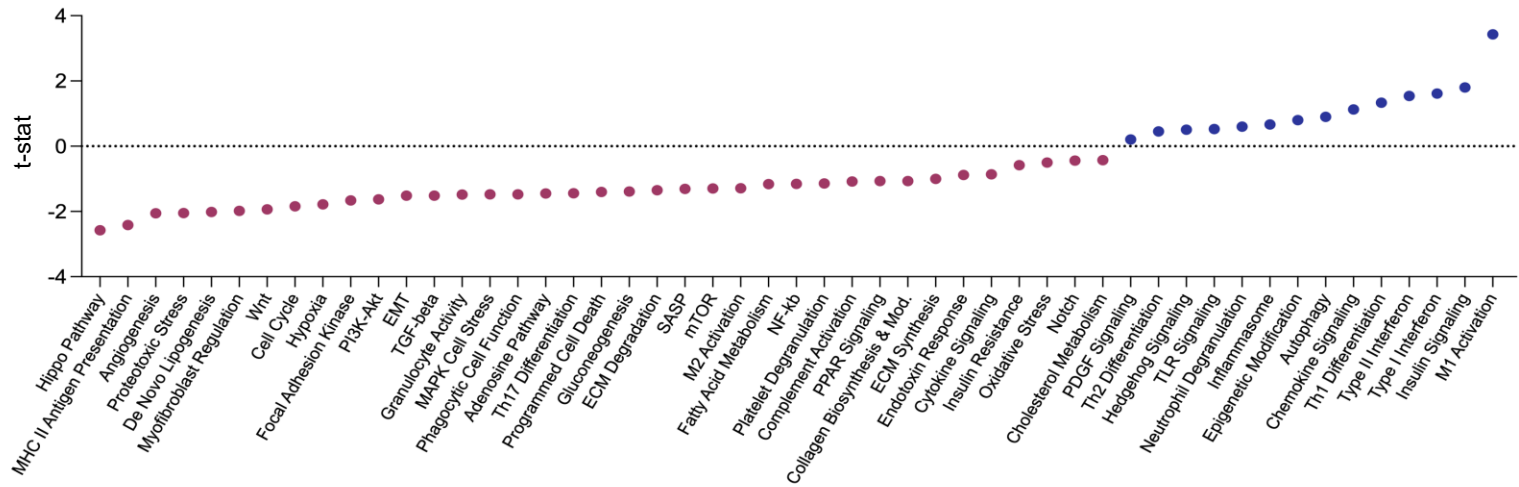

Supplement: szaf026_suppl_Supplementary_Figures_1-3 [file szaf026_suppl_supplementary_figures_1-3.pdf]
